# Interactive Effects of Root-Promoting Treatments and Media on Clonal Propagation of Two Western Pine Species

**DOI:** 10.3390/plants15020237

**Published:** 2026-01-13

**Authors:** Jaime Barros Silva Filho, Arnaldo R. Ferreira, Milton E. McGiffen

**Affiliations:** 1 US Forest Service, United States Department of Agriculture (USDA), Pacific Southwest Research Station, 4955 Canyon Crest Dr., Riverside, CA 92507, USA; 2 US Forest Service, United States Department of Agriculture (USDA), 1400 Independence Ave SW, Yates Building, Washington, DC 20250, USA; arnaldo.ferreira@usda.gov; 3 Department of Botany and Plant Sciences, University of California at Riverside, 900 University Avenue, Riverside, CA 92521, USA

**Keywords:** coconut coir, commercial mixed microbial/biostimulant, ponderosa pine, reforestation, rooted cuttings, sugar pine, vegetative propagation

## Abstract

Root-promoting treatments and media are critical for the clonal propagation of Western pine species, yet long-term data for sugar pine (*Pinus lambertiana* Dougl.) and ponderosa pine (*Pinus ponderosa* Dougl. ex C. Lawson) remain limited. This multi-year greenhouse study (2024–2025) used a split-plot greenhouse design to evaluate interactions among four rooting media and various root-promoting treatments, including commercial mixed microbial/biostimulant products and indole-3-butyric acid (IBA). Coconut coir consistently outperformed other media, achieving rooting rates of up to 80% when combined with specific commercial products like Mykos, Dynomyco, or Wildroot. Seasonal timing also proved crucial; winter propagation (January 2025) yielded poor results (<30%) regardless of treatment, consistent with the influence of physiological dormancy. While commercial mixed microbial/biostimulant products were associated with improved rooting, their effectiveness depended on the use of an appropriate substrate. The superior performance of coconut coir is consistent with its physical characteristics (e.g., aeration, water retention), although these properties were not directly measured.

## 1. Introduction

Sugar pine (*Pinus lambertiana* Dougl.) and ponderosa pine (*Pinus ponderosa* Dougl. ex C. Lawson) are two of the most ecologically and economically significant conifers in western North America [1,2]. Sugar pine is the tallest pine species, typically reaching 53–61 m in height and occasionally exceeding 80 m, with trunk diameters of 91–152 cm and lifespans that may span several centuries [1]. It provides the structural and biological underpinnings for the Sierra Nevada, the Cascade Range, and the coastal ranges of California and Oregon. Sugar pine was once prized for its straight-grained, lightweight wood, but today it is essential for the species it supports and for the long-lasting wood it provides in fire-prone areas [3]. Ponderosa pine is similarly widespread, occurring from British Columbia through the western United States into northern Mexico. It thrives in open, sunny environments and generally attains heights of 30–50 m and diameters of 0.6–1.3 m [2]. Its wood is widely used in construction and furniture manufacturing, and the species plays a central role in fire-adapted forest ecosystems [4].

Increasing occurrences of large wildfires, drought, and biotic stressors have substantially constrained the natural regeneration of these species, making seedling production a critical component of post-fire reforestation programs [5,6,7,8,9]. Seedlings typically require one to two years to reach plantable size, and microclimate, soil conditions, competition, and predation strongly shape their establishment success. For forests to be strong over the long term, it is important to keep genetic diversity across landscapes [10,11,12].

Within this context, clonal propagation is not intended to replace seed-based regeneration but rather to complement it. Clonal methods can keep high-value genotypes alive, make uniform planting stock for research or seed orchards, and quickly multiply individuals with desirable traits like disease resistance, stress tolerance, or faster growth [13,14,15,16]. Vegetative propagation enables the rapid multiplication of identical genotypes, a clear operational advantage, although this same uniformity necessarily reduces genetic diversity at the stand level [13,15]. As a result, clonal propagation is best considered as a targeted technique within larger, genetically varied regeneration initiatives [12,14,15].

Despite their ecological and managerial importance, there has been little research on clonal proliferation in sugar pine and ponderosa pine. Early research on sugar pine somatic embryogenesis reported substantial heterogeneity across genotypes and low conversion rates to viable plantlets, indicating the species’ resistance to vegetative propagation [17,18,19]. Rooting success in ponderosa pine stem cuttings has historically been low and variable, particularly when the donor material is more than a year old [20,21]. Temperature, humidity, photoperiod, and auxin administration all influence rooting responses, with different results reported in the literature [22]. These findings highlight a significant knowledge gap in the absence of scalable, reliable, and biologically informed clonal-propagation protocols for Western pine species.

Commercial mixed microbial/biostimulant products (many of which contain mycorrhizal species, biocontrol fungi, and other additives) have shown promise for improving pine seedling performance in nursery and field settings [23,24,25,26,27]. Furthermore, alternative substrates, such as coconut coir, may enhance root development; this pattern is consistent with improved aeration and moisture balance, although substrate physical properties were not quantified in this study. A thorough examination of the combined effects of rooting medium, commercial mixed microbial/biostimulant products, auxin treatments, and seasonal timing on the clonal proliferation of sugar pine and ponderosa pine remains lacking.

To overcome this gap, we investigated four hierarchical questions: (1) Substrate effect: Does coconut coir improve rooting performance over conventional substrates? (2) Inoculant effect: Do commercial mixed microbial/biostimulant products stimulate rooting or early root development? (3) The auxin effect: Does commercial IBA gel (Clonex) improve rooting success or increase taproot development? (4) Seasonal timing: Are there any changes in rooting effectiveness between cuttings obtained in January and March?

We hypothesized that using coconut coir with commercial mixed microbial/biostimulant products would yield better rooting success than traditional substrates and that auxin application and seasonal timing would also influence rooting responses in both species.

## 2. Materials and Methods

### 2.1. Location and Description

The studies were conducted in the US Forest Service Pacific Southwest Research Station greenhouse in Riverside, CA, USA (33°57′38.65″ N, 117°20′0.74″ W, 1109 feet or 338 m above sea level). To protect the unrooted cuttings from excessive transpiration and photoinhibition, the greenhouse was equipped with an aluminet shade cloth, which reduced the natural solar radiation to an average photosynthetic photon flux density (PPFD) of approximately 100 µmol m^−2^ s^−1^.

**Figure 1:**
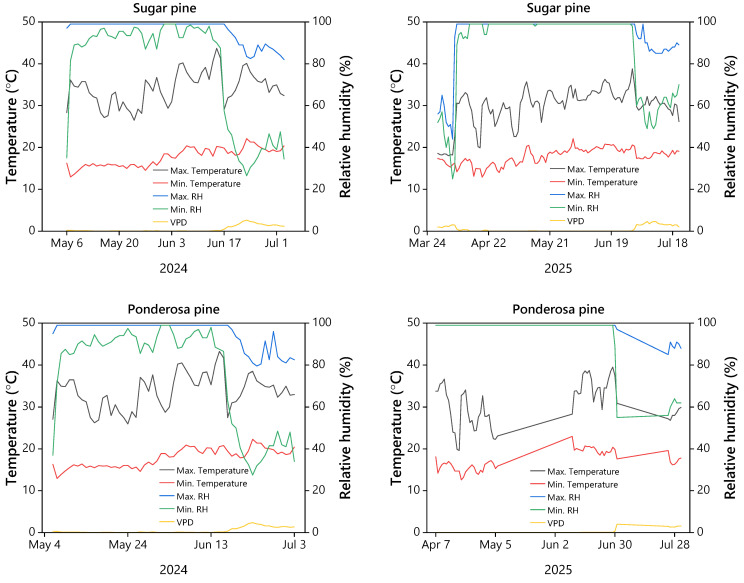
Daily averages of maximum and minimum temperatures, maximum and minimum relative humidity, and vapor pressure deficit (VPD) for sugar pine and ponderosa pine containers across the growing seasons in 2024 and 2025.

Environmental conditions were monitored continuously using SwitchBot indoor/outdoor thermo-hygrometers. To ensure the data reflected the actual microclimate experienced by the cuttings, a sensor was placed inside a representative 76 L (20-gallon) container in each experimental block. These sensors recorded the air temperature and relative humidity (RH) within the sealed “high-humidity” environment created by the 15 L of water at the base of the container. Daily maximum and minimum temperatures, RH, and calculated vapor pressure deficit (VPD) were extracted from these loggers for the duration of the 2024 and 2025 rooting seasons (Figure 1).

### 2.2. Studies

A 2022 preliminary study found that coconut fiber, when combined with tap water and without a rooting hormone, achieved a rooting success rate of 65%. The findings underscored the necessity to assess a wider variety of substrate and rooting-promoting treatment combinations. Consequently, the present study was initiated to examine the interactive effects of various rooting promoters and media on the propagation of cuttings in greenhouse conditions during the 2024 and 2025 seasons.

**Figure 2:**
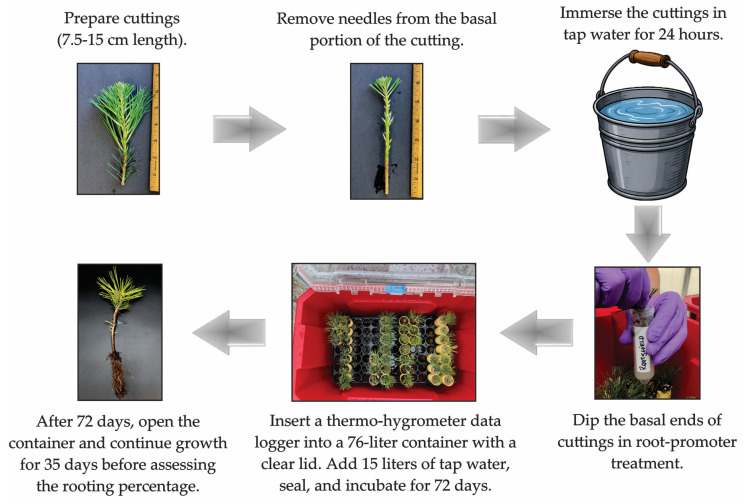
Flow diagram of the vegetative propagation process for sugar pine and ponderosa pine: from clipping to evaluation of rooted cuttings.

The propagation protocol was standardized across both years (Figure 2). To ensure consistent moisture availability, 15 L of tap water was added to the bottom of each 76 L (20-gallon) container prior to transplanting. This reservoir-maintained substrate moisture via capillary action throughout the experiment. The greenhouse environment was further stabilized by an evaporative cooling wall, which maintained moderate temperatures and high ambient humidity.

Cuttings (7.5–15 cm) were harvested from 1–2-year-old lignified stems, needles were removed from the lower two-thirds, and cuttings were immersed in tap water for 24 h. For all root-promoting treatments, the basal 3–4 cm of each cutting was immersed directly into the assigned product within 50 mL sterile centrifuge tubes for approximately 5 s. For the 0.31% IBA gel (Clonex) and all powdered or granular commercial mixed microbial/biostimulant products, materials were used undiluted, and no excess material was removed prior to planting.

Following treatment, cuttings were immediately planted into Ray Leach cells (66 cm^3^) and placed into the pre-watered containers. The containers were completely sealed for 72 days without manual ventilation to maintain a saturated-humidity environment. Lighting was provided by natural sunlight filtered through aluminet shade cloth, yielding a midday average PPFD of approximately 100 µmol m^−2^ s^−1^, measured inside the sealed containers at cutting height using a Sekonic C-7000 spectrometer. Natural photoperiods reflected ambient daylength at the greenhouse location, averaging approximately 13 h in March 2024, approximately 10 h in January 2025, and approximately 12 h in March 2025. After the 72-day sealed period, containers were opened and monitored for an additional 35 days. No supplemental watering was required during either phase, as the initial water reservoir and the greenhouse cooling system prevented media desiccation.

#### Donor Material

Cuttings for both species were collected from lignified stems (1–2 years old) of greenhouse-grown seedlings originating from multiple mother trees and a range of elevations. Donor identity was not tracked individually, as the study objective was to evaluate treatment and media effects across a broad genetic and provenance background. Cuttings from the mixed donor pool were randomly assigned across all root-promoting treatments and rooting media, ensuring balanced representation and avoiding bias toward any particular donor source. This process was designed so that donor-level variation would be distributed across experimental units and absorbed into the residual error term; however, individual donors were not tracked longitudinally across collection dates, so donor identity and seasonal effects cannot be fully disentangled in this study.

### 2.3. Design

The study utilized a completely randomized design (CRD) in a split-plot arrangement with 10 independent replications. The experiment was repeated over two years (2024 and 2025). The main plot experimental unit was the 76 L container (20-gallon), which received a single root-promoting treatment. Within each container, Ray Leach cells (66 cm^3^) were filled with four rooting media (coconut coir, sand, Promix MP, and Sunshine). For each species and container, 10 cells per medium were established, each cell containing one cutting, for a total of 40 cuttings per container per species (4 media × 10 cells). Thus, within each container, the four rooting media constituted the subplots, each represented by a group of 10 cells of the same medium. This structure resulted in 320 subplot experimental units in 2024 (8 treatments × 4 media × 10 containers) and 360 subplot experimental units in 2025 (9 treatments × 4 media × 10 containers).

While each container held 40 cuttings per species, statistical inference was based on the container level. Cuttings within each container × medium combination were treated as subsamples and aggregated to a single subplot value (either rooting percentage or mean taproot number) for analysis. Replication for treatment effects was defined as 10 containers per treatment, and replication for media and treatment × media effects was defined as 10 containers per medium within each treatment.

### 2.4. Root-Promoting Treatments Description

Seven treatments with root-promoting treatment plus an untreated control in 2024. For 2025, we added a Clonex treatment (0.31% IBA). Main-plot treatments are detailed in Table 1.

### 2.5. Rooting Media Description

We describe the subplot treatments in Table 2.

### 2.6. Data Collection

For each species, 107 days after transplanting, we recorded, for every Ray Leach cell, whether the cutting had formed roots (yes/no) and the number of taproots. A cutting was classified as ‘rooted’ only if it produced at least one root reaching a minimum length of 2 mm. Cuttings that exhibited only callus formation or root primordia <2 mm were considered unrooted. Only primary taproot axes were counted; secondary or lateral roots branching from the main axis were not included in taproot counts. For statistical analysis, data were aggregated at the container × medium level. Specifically, for each container × medium × species combination (10 cuttings), we calculated (i) the rooting percentage (number of rooted cuttings/10) and (ii) the mean number of taproots per cutting (total number of roots ≥ 2 mm/10).

### 2.7. Data Analysis

Data were analyzed independently for each year (2024 and 2025); no pooling across years was performed. The experimental design followed a completely randomized design (CRD) in a split-plot arrangement with 10 replications. The main plot factor was the root-promoting treatment, applied at the container level, and the subplot factor was the rooting medium, represented by the four media within each container. For each species, the experimental unit for treatment effects was the container; the experimental units for media and treatment × media interaction were the container × medium combinations, with the aggregated rooting percentage and mean taproot number per container × medium as response variables.

A split-plot analysis of variance (ANOVA) was conducted, using the container-level residual (Error a) as the error term for testing treatment effects and the subplot residual (Error b) for testing media and interaction effects. Residual diagnostics (normal probability plots and homogeneity of variance checks) confirmed approximate normality and homoscedasticity. Rooting percentage data were analyzed on the original scale. Rooting percentage data were analyzed on the original scale.

Post hoc comparisons were selected to balance statistical validity and interpretability. Rooting media means (subplot factor, 4 levels) were separated using Tukey’s HSD test (*p* < 0.05), which controls the family-wise error rate and is appropriate for balanced comparisons. Root-promoting treatment means (main plot factor, up to 9 levels) were grouped using the Scott-Knott ESD test (*p* < 0.05), chosen for its ability to form statistically distinct, non-overlapping clusters and to reduce Type I error inflation in multi-level comparisons.

Results are reported as estimated means ± standard errors. Effect sizes (partial η^2^) were calculated to provide context for biological relevance.

The formula below calculates partial eta-squared ($\eta_{p}^{2}$) effect sizes for any factor, including main plot, subplot, and interaction [28]:

$$\eta_{p}^{2}=\frac{{SS}_{factor}}{{SS}_{factor}+{SS}_{Error}}$$

where:

${SS}_{factor}$ is the sum of squares for the factor (root-promoting treatment, media, or their interaction).

${SS}_{Error}$ is the sum of squares for the residual (Error term).

All statistical analyses and graph outputs were performed using SAEG version 9.1 [29] and OriginPro (OriginLab Corporation, version 2025, Northampton, MA, USA) [30].

## 3. Results

### 3.1. Studies in March 2024 and 2025

Rooting performance in both sugar pine and ponderosa pine was primarily influenced by the choice of rooting medium. According to Tukey’s pairwise comparisons, coconut coir consistently outperformed sand, Promix MP, and Sunshine across both years.

**Figure 3:**
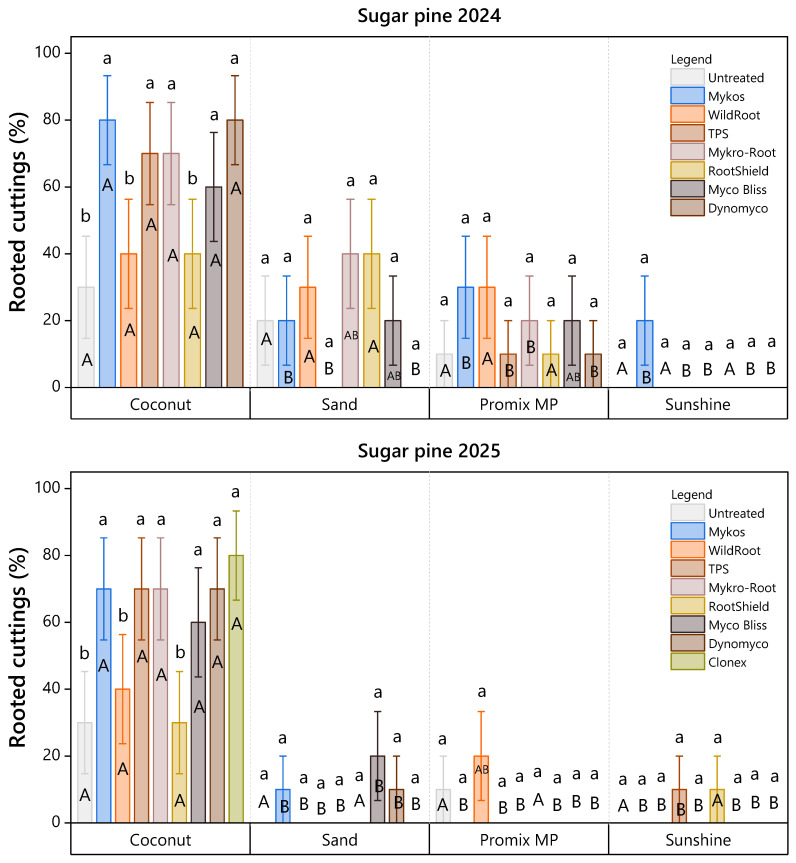
Rooting success of sugar pine cuttings (%) under eight treatments in 2024 and nine treatments in 2025 across four rooting media, assessed 107 days after planting. Lowercase letters indicate significant differences among root-promoting treatments within each medium (Scott-Knott ESD, *p* < 0.05), while uppercase letters indicate significant differences among rooting media within each root-promoting treatment (Tukey’s HSD, *p* < 0.05). Bars represent mean rooting percentages ± standard error. Each bar reflects *n* = 10 containers (experimental units), with 10 cuttings per substrate subplot aggregated to a single value per container.

For sugar pine in 2024, the rooting medium dictated the efficacy of root-promoting treatments. Within coconut coir, cluster analysis (Scott–Knott ESD) identified a high-performance group comprising Mykos (Xtreme Gardening, Valencia, CA, USA), TPS (TPS Nutrients, Spokane, WA, USA), Mykro-root (Microbial Applications, Inc., Suwanee, GA, USA), Myco Bliss (Plantonix, Albuquerque, NM, USA), and Dynomyco (AMY Living Soil, Tel Aviv, Israel), each achieving rooting rates of approximately 80%. These treatments were statistically distinct from the lower-performing group, which included the untreated control, Wildroot, and Rootshield. This pattern persisted in 2025, with Clonex gel containing IBA (Growth Technology Ltd., Taunton, Somerset, UK) clustering with the top-performing commercial mixed microbial/biostimulant products and showing no significant difference from them (Figure 3).

**Figure 4:**
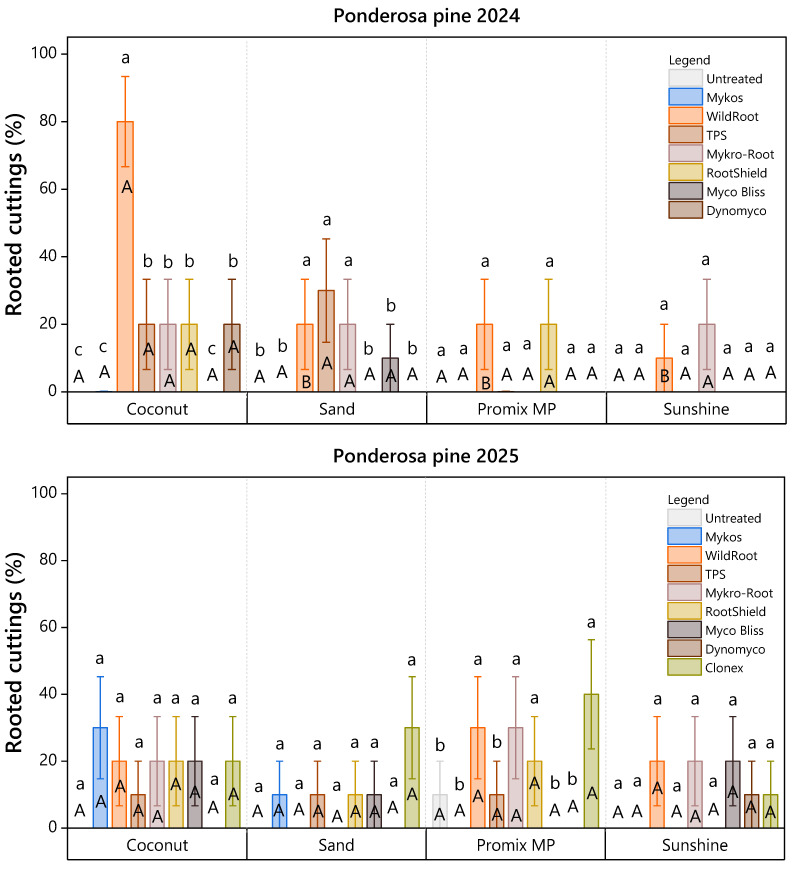
Rooting success of ponderosa pine cuttings (%) under eight treatments in 2024 and nine treatments in 2025 across four rooting media, assessed 107 days after planting. Lowercase letters indicate significant differences among root-promoting treatments within each medium (Scott-Knott ESD, *p* < 0.05), while uppercase letters indicate significant differences among rooting media within each root-promoting treatment (Tukey’s HSD, *p* < 0.05). Bars represent mean rooting percentages ± standard error. Each bar reflects *n* = 10 containers (experimental units), with 10 cuttings per substrate subplot aggregated to a single value per container.

Ponderosa pine exhibited greater interannual variability. In 2024, coconut coir remained the superior substrate, and Wildroot formed a unique statistical group within this medium, achieving significantly higher rooting success (80%) than all other treatments (Figure 4). However, overall success declined in 2025. While Wildroot, Mykro-root, Rootshield, and Clonex outperformed the control within Promix MP, the distinct advantage of coconut coir observed in the previous year was less pronounced.

**Figure 5:**
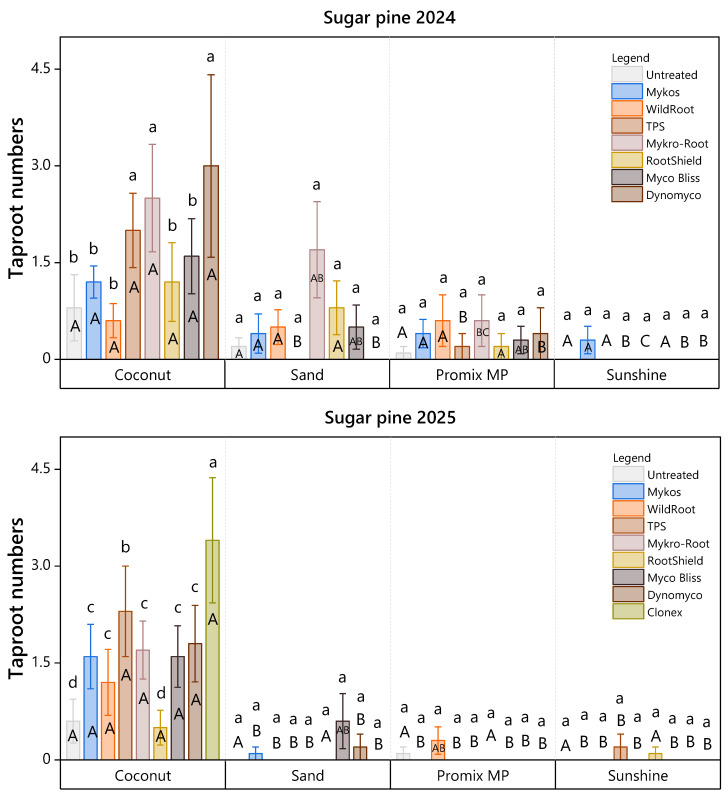
Taproot numbers of sugar pine cuttings under eight treatments in 2024 and nine treatments in 2025 across four rooting media were assessed 107 days after planting. Lowercase letters indicate significant differences among root-promoting treatments within each medium (Scott-Knott ESD, *p* < 0.05), while uppercase letters indicate significant differences among rooting media within each root-promoting treatment (Tukey’s HSD, *p* < 0.05). Bars represent mean taproot numbers ± standard error. Each bar reflects *n* = 10 containers (experimental units), with 10 cuttings per substrate subplot aggregated to a single value per container.

Taproot development followed a similar trend. In sugar pine (2024), TPS, Mykro-root, and Dynomyco clustered as the top-performing treatments for taproot numbers. In 2025, Clonex emerged as the most effective treatment, demonstrating statistical superiority over both the control and commercial mixed microbial/biostimulant products (Figure 5).

**Figure 6:**
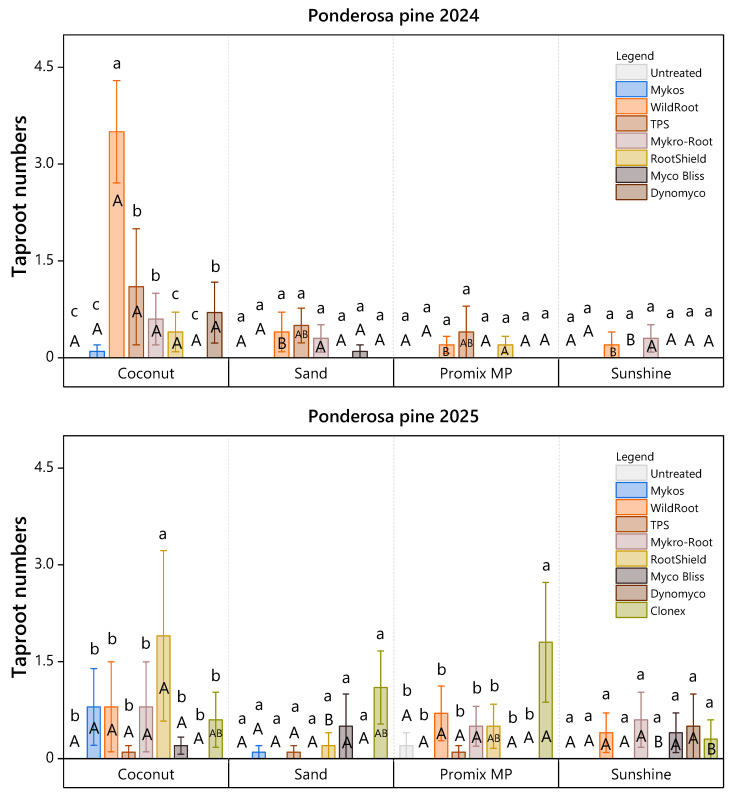
Taproot numbers of ponderosa pine cuttings under eight treatments in 2024 and nine treatments in 2025 across four rooting media, assessed 107 days after planting. Lowercase letters indicate significant differences among root-promoting treatments within each medium (Scott-Knott ESD, *p* < 0.05), while uppercase letters indicate significant differences among rooting media within each root-promoting treatment (Tukey’s HSD, *p* < 0.05). Bars represent mean taproot numbers ± standard error. Each bar reflects *n* = 10 containers (experimental units), with 10 cuttings per substrate subplot aggregated to a single value per container.

For ponderosa pine, Wildroot in coconut coir yielded the highest average taproot count in 2024 (3.2 ± 0.4), forming a statistical class superior to all other combinations. In contrast, cuttings propagated in sand consistently averaged fewer than 1.0 taproots, with no significant differences among treatments. By 2025, taproot formation declined overall, but Clonex (in Promix MP) and Mykro-root (in coconut coir) yielded the highest relative counts (Figure 6).

**Figure 7:**
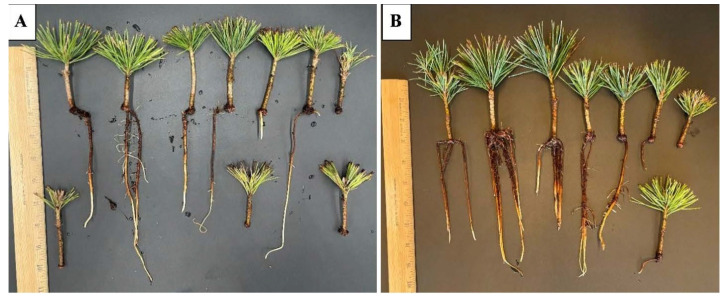
Rooted cuttings of sugar pine at 107 days after transplanting on coconut coir with Mykos (**A**) and Dynomyco (**B**) commercial root-promoting treatments in 2024.

In 2024, treatments with Mykos and Dynomyco yielded 80% rooted cuttings in sugar pine using coconut coir as the rooting medium (Figure 7A,B). However, in 2024, treatments with TPS and Mykro yielded 70% rooted cuttings in sugar pine using coconut coir as rooting media (Figure 3).

**Figure 8:**
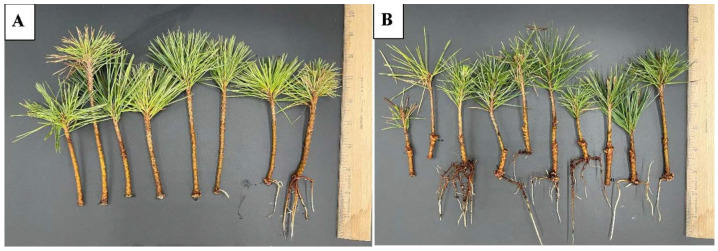
Rooted cuttings of sugar pine at 107 days after transplanting on coconut coir with Mykos commercial root-promoting treatment (**A**) and Clonex (**B**) treatments in 2025.

In 2025, treatments with Mykos and Clonex resulted in 70% and 80% rooted cuttings in sugar pine, respectively, using coconut coir as the rooting medium (Figure 8A,B).

**Figure 9:**
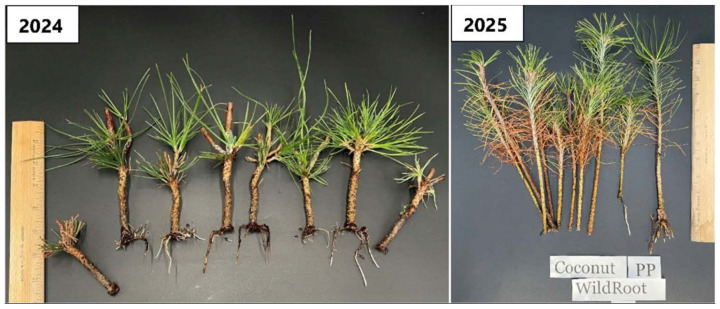
Rooted cuttings of ponderosa pine at 107 days after transplanting on coconut coir with Wildroot commercial root-promoting treatment in 2024 and 2025.

Treatment with Wildroot resulted in 80% and 20% rooted cuttings in ponderosa pine using coconut coir as the rooting medium in March 2024 and 2025, respectively (Figure 9: 2024, 2025).

### 3.2. Study in January 2025

The winter propagation study conducted in January 2025 yielded consistently poor results (<30%) across all treatments and media. These findings are consistent with the expectation that deep physiological dormancy in mid-winter reduces rooting competence, although we cannot exclude the contribution of differences in donor composition or physiological status between the January and March collections. Rooting success remained below 30%, and taproot development was similarly limited, with average counts falling below 1.0 for sugar pine and 0.5 for ponderosa pine.

Unlike in the spring trials, Tukey’s pairwise comparisons showed that the choice of rooting medium did not yield significant performance differences for most treatment combinations. Where significant effects did occur, they were largely confined to the coconut coir substrate. Within this medium, Scott–Knott cluster analysis revealed a reversal of the patterns observed in March: the untreated control frequently grouped within the highest-performing statistical category. For sugar pine, the control achieved approximately 10% success, statistically separating it from the eight commercial mixed microbial/biostimulant products, which rarely exceeded 2%. A similar pattern appeared in ponderosa pine, where the control reached roughly 30% success, forming a superior cluster compared to Myco Bliss (approximately 10%), while other commercial products remained statistically indistinguishable from the control.

Taproot data followed the same pattern. In coconut coir, the untreated controls for both species clustered in the top statistical tier, performing with equal or greater efficacy than the commercial treatments. In Sunshine, Promix MP, and sand, no significant differences among treatment clusters were detected for either species.

### 3.3. Effect Sizes for the Studies in March 2024 and 2025

Effect size analysis revealed a consistent hierarchy of influence across both species and years. As shown in Table 3, the rooting medium accounted for the largest proportion of variance in propagation outcomes, followed by the medium × treatment interaction, whereas the treatments alone contributed the least.

For sugar pine, the rooting medium explained a large proportion of the variance in rooting performance ($\eta_{p}^{2}$ = 0.427 in 2024; 0.528 in 2025). The interaction between medium and treatment had a moderate effect ($\eta_{p}^{2}$ = 0.174 and 0.158), but the main effect of treatment alone was still small ($\eta_{p}^{2}$ = 0.058 and 0.063).

Ponderosa pine showed the same overall hierarchy of effects. The rooting medium accounted for $\eta_{p}^{2}$ values of 0.252 in 2024 and 0.455 in 2025, while the interaction effect ranged from 0.133 to 0.202. The main treatment effect was moderate in magnitude ($\eta_{p}^{2}$ = 0.103 and 0.141).

## 4. Discussion

### 4.1. Studies in March 2024 and 2025

The majority of previous studies focused on loblolly pine (*Pinus taeda*), eastern white pine (*Pinus strobus* L.), jack pine (*Pinus banksiana*), masson pine (*Pinus massoniana*), and Fraser fir (*Abies fraseri*) [31,32,33,34,35,36,37,38,39]. In contrast, our multi-year greenhouse study on sugar pine highlighted the effectiveness of products containing the vesicular-arbuscular mycorrhizal fungus *Glomus intraradices* (syn. *Rhizophagus intraradices*), which consistently enhanced rooting performance. Despite the observation of notable rooting responses across several inoculant-medium combinations, microscopic verification of fungal colonization was not conducted in this study. Thus, the beneficial effects associated with commercial mixed microbial/biostimulant products should be regarded as biostimulatory responses at the rhizosphere level, such as increased nutrient mobilization, improved water relations, or signaling interactions, rather than as confirmed symbiotic colonization. The enhanced taproot growth observed with Clonex indicates a hormonal effect of exogenous auxin rather than direct evidence of endogenous auxin regulation. These results corroborate those of Scagel et al. [40], who investigated the effects of adding *Glomus intraradices* to the rooting substrate on Hick’s yew cuttings treated with a hormone solution (1.03% indole-3-butyric acid and 0.66% 1-naphthalene acetic acid). They found that incorporating *Glomus intraradices* into the substrate increased both root quantity and growth compared to using the hormone alone. Conversely, Scagel [41] examined the rooting of kinnikinnick (*Arctostaphylos uva-ursi* ‘Massachusetts’) hardwood cuttings using three different inoculants: *Glomus intraradices*, a hyphal inoculum from an arbutoid mycorrhizal fungus, and an inoculum composed of colonized root fragments of kinnikinnick. The aim was to evaluate whether adding this mycorrhizal fungus to the rooting media would enhance rooting and root growth during a period deemed unfavorable for rooting cuttings of this hardwood species. The author concluded that the application of *Glomus intraradices* did not affect rooting or root growth in kinnikinnick cuttings.

For ponderosa pine, the commercial treatments Wildroot and Myco Bliss have been shown to be the most effective. Both products include ectomycorrhizal fungus (*Rhizopogon* spp.) at varying concentrations: 16,500 propagules per gram in Wildroot and 35 propagules per gram in Myco Bliss. This suggests that the *Rhizopogon* species may enhance the rooting potential of this pine species. The decline in ponderosa pine rooting percentages from 2024 to 2025 (80% to 20%) when Wildroot was combined with coconut coir as a rooting medium may be due to the average size of the cutting used in 2025 (20 cm or 8 inches) being larger than that of the cutting used in 2024 (12.5 cm or 5 inches) [42]. Garcia et al. [43] examined the soil characteristics associated with the composition of ectomycorrhizal fungi in relation to ponderosa pine and lodgepole pine in the Deschutes National Forest, Oregon, USA. The authors found evidence that *Rhizopogon* spp. were dominant in soil samples from ponderosa pine and lodgepole pine.

In 2025, applying indole-3-butyric acid (IBA) at a concentration of 0.31% (Clonex) significantly improved the rooting success of sugar Pine (80%) and ponderosa Pine (40%) cuttings. This result aligns with broader propagation research showing that optimized auxin concentrations enhance adventitious root formation and improve root system morphology in stem cuttings [44]. The use of IBA as an auxin has been shown to increase rooting rates significantly, e.g., research involving cuttings (10–12 cm or 4–5 inches) immersed in a 0.1% IBA Benlate-95% ethyl alcohol solution for five seconds can achieve a 60% rooting success rate in Eastern White Pine (*Pinus strobus* L.) [45]. Parladé et al. [46] indicate that some fungi promote root branching during mycorrhizal colonization by producing varying quantities of indole-acetic acid (IAA).

Factors that may influence rooting success include plant growth regulators, light quality, temperature, and the properties of the rooting media. These factors require further refinement to develop reliable techniques for adventitious rooting in conifers [22,47,48]. The age, diameter, length, and physiological condition of the donor tree’s cuttings, along with environmental factors such as humidity and moisture availability, may significantly affect root development. Higher humidity and low moisture stress can be essential for the formation of strong roots [21,49,50].

This study did not directly measure auxin concentrations, glucose levels, or mycorrhizal colonization; nonetheless, the observed treatment responses are consistent with mechanisms proposed in previous research. Mycorrhizal inoculation correlates with increased auxin production and improved carbohydrate transport, notably higher glucose availability, as evidenced by prior research [51,52,53,54]. Coconut coir enhances aeration and moisture balance owing to its significant water-holding capacity and structural properties [55,56]. Thus, these explanations should be treated as hypotheses based on the existing literature, rather than as direct physiological measurements from this experiment. The enhanced rooting success associated with commercial mixed microbial/biostimulant products and coconut coir rooting medium may be attributed to multiple physiological mechanisms. Auxin transport and sensitivity may control adventitious root growth in conifer stem cuttings [57]. Mycorrhizal fungi may influence auxin metabolism by either synthesizing auxin analogs or enhancing endogenous auxin production, thereby facilitating cell dedifferentiation and the initiation of root primordia [58,59]. Infected cuttings also tend to have higher glucose levels. Mycorrhizae improve photosynthetic efficiency and facilitate the transport of soluble carbohydrates, which serve as energy sources and building blocks for root growth [59]. Mature cuttings, possessing partially lignified tissues, generally exhibit diminished rooting efficiency due to decreased auxin transport capacity and lower carbohydrate reserves; nonetheless, inoculation may mitigate these constraints by augmenting sink strength at the basal stem region and enhancing nutrient absorption [60]. The persistent benefit of coconut coir as a rooting medium is likely due to its substantial water retention and aeration, which sustain optimal oxygen and moisture levels for fungal colonization and root development. These physiological interactions indicate that effective clonal multiplication relies on both substrate characteristics and the synergistic influence of mycorrhizal symbiosis on hormone signaling and glucose metabolism in growing cuttings. Collectively, these findings indicate that the efficacy of commercial mixed microbial/biostimulant products is contingent upon context and influenced by substrates, species, and physiological conditions during propagation.

Although coconut coir consistently outperformed other substrates, this study did not quantify substrate physical properties, such as bulk density, air-filled porosity, water-holding capacity, pH, or electrical conductivity. These unmeasured variables likely contributed to the observed differences among media, particularly under the sealed water reservoir conditions used here. The superior rooting performance observed in coir is therefore best interpreted as consistent with favorable physical characteristics, rather than as a confirmed mechanistic outcome. Future studies should include basic substrate characterization to clarify the role of media properties in rooting success.

### 4.2. Study in January 2025

Rooting success in January 2025 was low (approximately 30%), likely due to deep physiological dormancy and unfavorable season conditions. According to Wise and Caldwell [61], species exhibit varying degrees of rooting recalcitrance; however, this inherent ability is heavily modulated by seasonal phenology. The significant disparity between our March trials (up to 80% success) and January trials is consistent with a strong influence of the dormancy cycle on rooting competence. Because individual donors were not tracked and resampled across months, these seasonal patterns necessarily integrate both phenological state and any shifts in donor composition or physiological condition between collection dates and should therefore be interpreted as operational rather than strictly mechanistic seasonal effects. Although our results clearly show that January cuttings rooted poorly compared with March cuttings, these data represent only a single winter–spring comparison. When considered alongside external observations reporting low rooting success for cuttings collected between July and January, early spring appears to be a more favorable period for collection; however, broader seasonal trials are needed to confirm this pattern. This seasonal effect is widely documented in North American conifers; rooting competence typically peaks in early spring when the transition out of endodormancy coincides with rising endogenous auxin levels and the reactivation of the cambium [61,62,63]. Conversely, rooting potential is frequently restricted during the summer and fall as cuttings transition into lignification and deep dormancy. While previous observations (e.g., Silva Filho [42]) noted low rooting rates (10–30%) for collections from July to January, our findings provide direct experimental evidence that early spring represents a superior collection window for sugar and ponderosa pine. However, as this study did not include year-round sampling, the hypothesis that March and April represent the absolute optimal window remains a preliminary conclusion that requires further verification across the full annual cycle.

While our results clearly support early spring as a superior collection window, the seasonal contrast observed here necessarily reflects both the phenological state and the donor pool available at each time point. Because donor trees were not tagged and resampled across months, donor-level variation is absorbed into the residual term of the split-plot ANOVA. This design is appropriate for estimating average operational responses across heterogeneous nursery stock (our primary objective), but it limits the strength of mechanistic seasonal inference. Future studies that track individual donors across multiple collection windows would allow a more explicit separation of donor, seasonal, and treatment effects and provide a clearer understanding of rooting dynamics across the annual cycle.

### 4.3. Effect Sizes for the Studies in March 2024 and 2025

To interpret the magnitude of the experimental effects, we utilized partial eta-squared ($\eta_{p}^{2}$). Because no widely accepted domain-specific thresholds exist for interpreting partial eta-squared in plant propagation studies, we followed Cohen’s general guidelines for small, medium, and large effects. These benchmarks were considered in light of the meta-analysis by Barker et al., who reported generalized eta-squared values for rooting traits in Salicaceae. Although generalized η^2^ and partial η^2^ are related, they are not identical measures; therefore, the Barker study provides a useful context but cannot be treated as a direct benchmark for the effect sizes reported here. It is important to note that while $\eta_{p}^{2}$ and $\eta_{G}^{2}$ are related measures of effect size, they are not identical; $\eta_{p}^{2}$ calculates the proportion of variance associated with a factor, excluding other factors from the denominator, whereas $\eta_{G}^{2}$ is often preferred in meta-analyses for its comparability across different experimental designs. Consequently, the Barker et al. study is treated here as a broader context for effect magnitude in forest species rather than a direct numerical benchmark. Barker et al. [64] conducted a meta-analysis of 352 sets of genetic, environment, and genotype-by-environment variation estimates from 72 Salicaceae studies to assess sources of variation across plant, insect, and environmental characteristics. The authors used the generalized eta-squared (η^2^) effect size metric to measure the proportions of variation explained by plant genotype (G), environment treatment (E), and their interaction (G × E), providing a comparable framework for interpreting partial eta-squared.

The analysis of effect sizes provides critical insight into the factors that drive the success of vegetative propagation of Western pine cuttings. The results demonstrate that the rooting media had the strongest impact on rooting performance for both sugar pine and ponderosa pine in both years. The large partial eta-squared values provide evidence for this ($\eta_{p}^{2}$ > 0.4 in three out of four cases), far exceeding the effect of the root-promoting treatments themselves. This indicates that while both factors contribute to success, the substrate’s physical and chemical properties—such as aeration and water retention—exert a primary influence on rooting outcomes. In this context, the rooting medium may be regarded as a foundational requirement, providing the necessary environment for exogenous hormones or biological products to effectively exert their biochemical effects [65,66]. These findings are consistent with earlier research on conifer propagation, which often identifies maintaining optimal moisture and oxygen levels in the root zone as the principal environmental requirement [67]. Overall, the rooting medium exerted a stronger and more consistent influence on rooting success than any individual biological or hormonal treatment. While media selection appears to play a primary role in determining rooting outcomes under the conditions tested here, commercial mixed microbial/biostimulant products and auxin treatments still contributed meaningful improvements within favorable substrates.

Furthermore, the statistically significant, medium-to-large interaction effects ($\eta_{p}^{2}$ ranging from 0.133 to 0.202) confirm that the efficacy of root-promoting treatments is closely contingent upon the rooting medium in which they are applied. These interactions indicate that media and therapies work together rather than in opposition. For example, coconut coir is a better medium for drainage and aeration, which means it likely provides the aerobic conditions required for the metabolic processes that initiate root promotion. On the other hand, an unsuitable medium might cancel the advantageous benefits of a pharmacological stimulant since it causes anoxic stress.

The consistently small to medium effect sizes observed for the main root-promoting factor (ranging from $\eta_{p}^{2}$ = 0.058 to 0.141) indicate that, while the treatments did produce statistically significant differences in some specific cases (as shown in the earlier results discussion), the overall variance explained by this factor alone is limited. Root-promoting substances, including auxins (IBA, IAA, and NAA), are widely used to improve rooting success; however, their efficacy depends on optimal environmental and media conditions. Ludwig-Müller et al. [68] assert that auxins, including indole-3-butyric acid (IBA) and 1-naphthaleneacetic acid (NAA), enhance rooting percentage, increase the quantity of roots per cutting, improve rooting uniformity, and facilitate rooting in challenging species when applied with suitable propagation techniques. This highlights their role as auxiliary support rather than a principal driver of rooting. The higher effect size observed for the root-promoting treatments in ponderosa pine ($\eta_{p}^{2}$ approx. 0.14) compared to sugar pine ($\eta_{p}^{2}$ approx. 0.06) suggests that ponderosa pine cuttings may be more physiologically responsive to external stimulants. This likely reflects inherent differences in rooting recalcitrance between the two species; sugar pine has historically been documented as more challenging for vegetative propagation. While both species benefit from treatments, the greater sensitivity in ponderosa pine suggests a higher degree of physiological plasticity in response to exogenous auxins and commercial mixed microbial/biostimulant products. However, as endogenous hormone concentrations were not quantified, the specific biochemical pathways responsible for this differential responsiveness remain speculative.

The superior aeration and moisture retention of coconut coir are prerequisites for root-promoting agents to function effectively. Although substrate physical properties such as air-filled porosity and oxygen diffusion rates were not measured, we hypothesize that coir provided an optimal physical environment that facilitated the metabolic processes required for root initiation.

Ponderosa pine exhibited slightly larger effect sizes for root-promoting treatments than sugar pine. This pattern may indicate that ponderosa pine is inherently more responsive to exogenous biological or hormonal stimulation during early root initiation. However, the magnitude of the difference in this study remains modest. Previous work has similarly reported species-specific variation in responsiveness to auxin and commercial mixed microbial/biostimulant products among conifers, suggesting that physiological rooting capacity is not uniform across taxa.

## 5. Conclusions

Our study demonstrates that rooting success in sugar pine and ponderosa pine cuttings is primarily determined by the physical properties of the rooting medium, with coconut coir consistently outperforming other substrates across years and species. In addition, it indicates that coconut coir serves as a superior rooting medium compared to peat-based or sand alternatives, consistently yielding optimal rooting percentages and taproot counts. Commercial mixed microbial/biostimulant products, Mykos, Dynomyco, and Clonex, may substantially improve rooting; however, their effectiveness depends on the use of adequate rooting media. Therefore, the success of clonal propagation for Western pine species is strongly influenced by the time of year at which cuttings are collected. For example, propagation that started in March worked, but attempts that began in January yielded poor results, likely due to the cuttings being in a state of deep physiological dormancy.

Together, these findings may establish a reproducible framework for optimizing clonal propagation protocols in Western pine species. Prioritizing the selection of rooting media is crucial for successful propagation, while commercial mixed microbial/biostimulant products and auxin-based treatments can be strategically incorporated to improve rooting during optimal seasonal conditions.

This research indicates that the choice of an appropriate rooting medium (particularly coconut coir) and the timing of collection are the most important factors influencing rooting success in sugar pine and ponderosa pine. Commercial mixed microbial/biostimulant products and auxin treatments can improve root growth, but their effectiveness is strongly influenced by the time of year at which cuttings are collected. For instance, while collections in March yielded success rates up to 80%, January trials were largely unsuccessful due to deep physiological dormancy. Furthermore, the efficacy of root-promoting agents is contingent upon the substrate’s physical conditions. While these findings provide a solid foundation for improving Western pine clonal propagation, it is crucial to note that they are based on controlled greenhouse studies. Further research is required to validate the scalability of these techniques in large nursery operations and to investigate how environmental factors, such as light quality, influence long-term field performance. Because colonization was not assessed, these effects cannot be attributed specifically to mycorrhizal symbiosis.

These recommendations are based on controlled greenhouse experiments with two Western pine species under a specific set of environmental conditions. Field performance, long-term survival, and operational scalability remain to be evaluated before broad application in reforestation programs.

## 6. Limitations and Future Directions

This research examined the rooting success and taproot growth of sugar pine and ponderosa pine cuttings under controlled greenhouse conditions. Although these data provide important insights into the effects of commercial mixed microbial/biostimulant products and rooting media, we did not evaluate the survival, growth, or establishment of rooted cuttings after transplantation to the field. For reforestation to succeed, species must persist in their natural habitats over the long term, including having the ability to withstand drought, resist disease, and compete with other vegetation. Future research should therefore extend beyond greenhouse rooting trials to assess field performance, survival rates, and growth trajectories, thereby linking propagation methods to practical outcomes in restoration forestry.

We recommend conducting a study on the rooting of cuttings every month, starting in February and continuing through June, for both species (US Western pine species). Furthermore, it is necessary to explore transitioning from the current model of rooted cuttings in containers to a bench system that incorporates a fog system with a misting nozzle for commercial production. Future research should investigate how different light sources—such as LEDs, fluorescent lighting, or natural sunlight—affect rooting responses under controlled conditions, particularly when combined with commercial mixed microbial/biostimulant products and auxin products. Seasonal timing also appears to be an important factor in rooting success, and additional trials conducted across the full annual cycle would help identify the most favorable periods for collecting Western pine cuttings.

A further limitation is that donor identity was not tracked across collection dates, which prevented us from formally separating seasonal phenology from potential differences in donor composition or physiological status between January and March; as a result, the seasonal patterns reported here should be interpreted as operational outcomes within the donor pool available at each time point rather than as fully disentangled causal effects of season alone.

## Figures and Tables

**Table 1:** Root-promoting treatments described for the rooting of Western pine stem cuttings.

Treatment	Main Species/Strains	Concentration/Propagules	Additional Components
**Mykos ^1^**	*Rhizophagus intraradices*	300 propagules g^−1^	–
**Wildroot ^2^**	**Nine endomycorrhizal species**:		–
	*Glomus intraradices*	69 propagules g^−1^	
	*Glomus mosseae*	69 propagules g^−1^	
	*Glomus aggregatum*	69 propagules g^−1^	
	*Glomus etunicatum*	69 propagules g^−1^	
	*Glomus monosporum*	34 propagules g^−1^	
	*Glomus deserticula*	34 propagules g^−1^	
	*Glomus clarum*	34 propagules g^−1^	
	*Paraglomus brasilianum*	34 propagules g^−1^	
	*Gigaspora margarita*	34 propagules g^−1^	
	**Seven ectomycorrhizal species**:		
	*Rhizopogon villosulus*	16,500 propagules g^−1^	
	*Rhizopogon luteolus*	16,500 propagules g^−1^	
	*Rhizopogon amylopogon*	16,500 propagules g^−1^	
	*Rhizopogon fulvigleba*	16,500 propagules g^−1^	
	*Pisolithus tinctorius*	220,000 propagules g^−1^	
	*Scleroderma cepa*	11,000 propagules g^−1^	
	*Scleroderma citrinum*	11,000 propagules g^−1^	
**TPS ^3^**	**Four endomycorrhiza species**:	4–6 colony-forming units g^−1^ each	*Bacillus* spp. (5 species) and *Trichoderma* *harzianum*
	*Glomus intraradices*		
	*Glomus mosseae*		
	*Glomus aggregatum*		
	*Glomus etunicatum*		
**Mykro-root ^4^**	*Trichoderma harzianum*, *Trichoderma viride*	2 × 10^7^ colony-forming units g^−1^	Micronutrients (Zn, Cu, Mn, Mg, and Fe)
**Rootshield Plus WP ^5^**	*Trichoderma harzianum*	1.15%	–
	*Trichoderma virens strain* G-41	0.61%	
**Myco Bliss ^6^**	**Four ectomycorrhiza species**:	35 propagules g^−1^ each	–
	*Rhizophagus irregularis*		
	*Rhizophagus aggregatus*		
	*Rhizophagus proliferum*		
	*Rhizophagus clarus*		
	and *Claroideoglomus*		
	*etunicatum*		
**Dynomyco ^7^**	*Glomus intraradices*	700 propagules g^−1^	–
	*Glomus mosseae*	200 propagules g^−1^	
**Clonex (2025 only) ^8^**	IBA (Indole-3-butyric acid)	0.31%	Rooting gel formulation

^1^ https://www.xtreme-gardening.com/mykos-mycorrhizae (accessed on 6 January 2026), ^2^ https://wildrootorganic.com/ (accessed on 6 January 2026), ^3^ https://tpsnutrients.com/products/billions (accessed on 6 January 2026), ^4^ https://mikrobs.com/products/mikro-root-2oz (accessed on 6 January 2026), ^5^ https://bioworksinc.com/products/rootshield/ (accessed on 6 January 2026), ^6^ https://www.plantonix.com/products/myco-bliss (accessed on 6 January 2026), ^7^ https://www.dynomyco.com/ (accessed on 6 January 2026), and ^8^ https://www.hydrodynamicsintl.com/product/clonex-rooting-gel/ (accessed on 6 January 2026).

**Table 2:** Characteristics of rooting media used in clonal propagation of two Western pine species.

Rooting Media	Composition	Mycorrhizal Inoculation	Key Properties
**Coconut coir ^1^**	Processed coir fiber	None	High water-holding capacity, good aeration
**All-purpose Sand ^2^**	Coarse processed sand (washed)	None	High drainage, low nutrient retention
**Pro Mix MP ^3^**	sphagnum peat moss (60–70% by volume), coir, perlite, limestone, and wetting agent	*Glomus intraradices* (1 viable spore g^−1^)	Enhanced nutrient uptake, organic formulation
**Sunshine Mix #4 ^4^**	Canadian sphagnum peat moss, coarse perlite, coir pith (coir), gypsum, dolomitic limestone, wood biochar, and wetting agent	Endomycorrhiza strains included	Balanced moisture retention and aeration

^1^ https://vermontorganics.com/products/ (accessed on 6 January 2026), ^2^ https://www.quikrete.com/productlines/sandallpurpose.asp (accessed on 6 January 2026), ^3^ https://www.greenislanddistributors.com/product/pro-mix-mp-mycorrhizae-organik/ (accessed on 6 January 2026), ^4^ https://sunshinemixes.com/product/sunshine-mix-4-aggregate-plus-with-mycorrhizae/ (accessed on 6 January 2026).

**Table 3:** Partial eta squared ($\eta_{p}^{2}$) effect sizes for rooting performance, comparing root-promoting treatments and media across two Western pine species over two years.

Factors	2024	2025
**Sugar pine**		
Root-promoting treatment $\eta_{p}^{2}$	0.058	0.063
Media $\eta_{p}^{2}$	0.427	0.528
Interaction $\eta_{p}^{2}$	0.174	0.158
**Ponderosa pine**		
Root-promoting treatment $\eta_{p}^{2}$	0.103	0.141
Media $\eta_{p}^{2}$	0.252	0.455
Interaction $\eta_{p}^{2}$	0.133	0.202

## Data Availability

The new data generated here were created solely for testing our treatments and hypotheses and are not intended to be entered into any public database or archive. Thus, data sharing does not apply to this article.
